# Early behavioral indicators of aberrant feces in newly-weaned piglets

**DOI:** 10.1186/s40813-024-00396-4

**Published:** 2024-11-05

**Authors:** Vivian L. Witjes, Fleur Veldkamp, Francisca C. Velkers, Ingrid C. de Jong, Ellen Meijer, Johanna M. J. Rebel, Jan A. Stegeman, Tijs J. Tobias

**Affiliations:** 1 Department Population Health Sciences, Veterinary Medicine, Farm Animal Health, Utrecht University, Yalelaan 7, 3584 CL Utrecht, The Netherlands; 2 Department Animal Welfare and Health, Wageningen Livestock Research, Wageningen University and Research, De Elst 1, 6700 AH Wageningen, The Netherlands; 3 Adaptation Physiology Group, Wageningen University and Research, Wageningen, 6700 AH The Netherlands; 4 Behavior and Welfare Group, Department Population Health Sciences, Faculty of Veterinary Medicine, Utrecht University, Yalelaan 107, NL-3584 CL Utrecht, The Netherlands; 5 Royal GD, Arnsbergstraat 7, 3718EZ Deventer, The Netherlands; 6 Wageningen Bioveterinary Research, Wageningen University and Research, Houtribweg 39, 8221 RA Lelystad, The Netherlands

**Keywords:** Post-weaning diarrhea, Behavioral activity, Automated detection, Targeted intervention, Early indicators

## Abstract

**Background:**

Post-weaning diarrhea (PWD) is a frequently occurring health and welfare issue in weaned piglets. Behavioral changes indicating impaired health may be detectable before the onset of signs and could be useful to detect the development of PWD early, enabling targeted and timely interventions. Current algorithms enable automated behavioral classification on the group level, while PWD may not affect all piglets in one pen and individual level analysis may be required. Therefore, this study aimed to assess whether changes in pen activity or individual piglet behavior can be early indicators of the occurrence of PWD. During 3 replicated rounds, 72 piglets (*Sus scrofa domestica*, Landrace x Large White) weaned at 27 days of age, were housed in 4 pens with 6 piglets each. Individual fecal color and consistency were scored (0–5; ≥ 3 considered as aberrant feces) six times during the first two weeks post-weaning using rectal swabs. Additionally, using a similar scoring scale, feces on the pen floor were assessed daily. Two methods were applied for behavioral scoring. Individual behaviors (eating, drinking, standing, walking; n = 48) were scored manually and instantaneously with a five-minute interval from videos of the first two rounds, while pen activity (eating, drinking, moving; n = 12) was analyzed automatically and continuously using a commercially available algorithm from videos of all three rounds.

**Results:**

Piglets showing a relatively higher proportion of standing behavior one day before fecal scoring had increased odds of an aberrant fecal color score (odds ratio (OR): 4.8; 95% confidence interval (CI): 1.5–15.3). Furthermore, odds of aberrant colored feces increased in pens where piglets showed more moving activity two days before (OR: 6.14; 1.26 < 95%CI < 29.84), which was also found for fecal consistency (OR: 4.77; 95%CI: 1.1–21.6).

**Conclusions:**

Our results indicate that increased standing in individual piglets and an increased moving activity on the pen level may be important behavioral indicators of PWD before the onset of diarrhea. Further development of current algorithms that can identify behavioral abnormalities in groups, from the pen to the individual level, may therefore be a promising avenue for improved and targeted health and welfare monitoring.

**Supplementary Information:**

The online version contains supplementary material available at 10.1186/s40813-024-00396-4.

## Background

Studies have shown different associations between behavior and disease in animals such as pigs, also termed ‘sickness behavior’ [21]. Subtle behavioral changes may occur before the onset of other clinical signs and could therefore function as an early indicator. For example, changes in activity levels and feed and water intake may occur over time which could be important early indicators of reduced health and welfare in pigs [5, 55]. Advancements in the field of precision livestock farming have led to the development of automated sensor systems and associated algorithms, allowing researchers and stakeholders to collect continuous data on pig activity behaviors, among other data collections related to health and welfare [3]. The automatically collected data can be especially suitable for combining ethological observations with disease and welfare monitoring [38].

More specifically, *Salmonella* infected pigs show a reduction in feeding and drinking activity [1], while they may spend more time in ventral recumbency, sitting and standing than unaffected pigs [44]. Miller et al. [39] showed that, after a vaccination challenge, early behavioral changes in the pen compared to non-vaccinated controls included decreased standing, increased lying, and altered feeding and drinking rates. Taken together, pigs behaviorally respond in various ways to different disease challenges and behavioral changes may function as early signals of impaired health within a group. In addition, besides regular clinical observations which can cause a flight-fright response in pigs due to human presence and observation biases, continuous and automatic detection of activity behaviors and postures in practice may aid in a more accurate and early detection of impaired health.

A highly prevalent disease in pig production, causing mortality in newly weaned piglets, is post-weaning diarrhea (PWD) [29, 47]. Here, we define the occurrence of PWD when a piglet or pen is showing aberrant feces during the first two weeks post-weaning, meaning fecal matter of a looser consistency or an abnormal color [16, 32]. During the abrupt weaning process, piglets are confronted with a variety of stressors and risk factors including separation from the sow and siblings, social mixing, handling and transport, a novel housing environment, different climatic conditions and an increased risk of pathogen exposure [23, 53, 54]. Together with a changed diet, these circumstances increase chances of the development of diarrhea and dehydration, which may ultimately even lead to death [8, 27, 33, 41, 47].

Besides these many different risk factors related to weaning, stress resilience and immune competence of individual piglets, and different pathogens (e.g. enterotoxigenic or enteropathogenic *Escherichia coli, rotavirus*) with different virulence factors (e.g. F4, F16) may be associated with PWD. Consequently, the etiology of PWD is complex, and the presence of a specific pathogen in fecal matter post-weaning may not always indicate the development of PWD. Moreover, not all individuals may be affected at the same time or in the same pen. Instead of pathogen detection, more general early behavioral changes, including altered feeding, drinking or activity patterns, before the onset of diarrhea may be especially useful as early indicators of PWD. Madsen and Kristensen [35] developed a tool based on water intake for predicting the occurrence of pen level PWD and Kyriazakis et al. [28] recently found that drinking behaviors increased during the first six days post-weaning in pens that had loose feces compared to pens with normal feces.

Currently, farmers and veterinarians can identify the occurrence and prevalence of PWD on the pen level by monitoring the appearance of feces, including consistency and color [16, 32] and soiling of the hind legs [17]. However, piglets are then already suffering from diarrhea, while specific and subtle changes in activity behaviors on the individual level may help farmers to early identify PWD in specific piglets. This may allow more timely interventions such as antimicrobial treatment on individual pig level instead of pen or barn level, and eliminating risk factors around weaning to prevent other piglets to fall ill. Thus, to target piglets showing early signs of PWD more efficiently, behavioral monitoring during the first week post-weaning on the individual level may be helpful to reduce PWD prevalence and antimicrobial use, while currently available algorithms primarily focus on the pen level.

Therefore, the aim of this study was to establish whether individual- or pen level changes in activity behaviors can be used as early indicators of aberrant fecal color or consistency in newly weaned piglets. We hypothesized that piglets showing aberrant feces, would show less movement and feeding activity one or two days before the onset of diarrhea, compared to conspecifics with normal stools. Data was collected on a total of 72 piglets, during three replicated rounds, with 24 piglets per replicate from the day of weaning until 12 days post-weaning. The presence of aberrant feces indicating PWD was determined by scoring rectal swabs [16, 19] or scoring feces from the pen floor. Behaviors were scored by either one of two methods: 1) instantaneous sampling on the individual level and 2) continuous scoring by an automated algorithm on the pen level. Besides using the algorithm, it was explored whether activity output on the pen level as measured by passive infrared detectors (PIDs,Veldkamp et al., in preparation; [51, 52]) could be used as an early indicator of PWD.

## Materials and methods

### Animals, housing and management

This study was performed at a semi-commercial pig farming facility in the Netherlands where weaning diarrhea frequently occurred spontaneously. The farm functions both as a commercial farm and a site for performing feeding trials. For this purpose, piglets are housed in relatively small pens, with an average of six weaned piglets per pen. Data collections were conducted during regular feeding trials performed at the facility simultaneously. During a pilot study conducted six weeks before data collection started, a virulent strain of *Escherichia coli* (F4 +) was detected in fecal samples, indicating the presence of a possible causal pathogen for PWD at the farm. Data was collected from four pens for three consecutive replicated rounds. The piglets (*Sus scrofa domestica*, Landrace x Large White crossbred bred with semen from a synthetic Large White boar line) were born from a total of 36 sows (round 1: 8; round 2: 13 and round 3: 15 sows) with a mean parity of 4.4 (round 1: 5.8; round 2: 4.5 and round 3: 3.0) and 5 first parity sows (round 1: 6 piglets from 1 first parity sow; round 2: 7 piglets from 4 first parity sows and round 3: no first parity sows) and ear tagged within 24 h after birth. Before weaning, piglets were supplemented with different diets of pelleted feed and/or a milk replacer across replicated rounds (see Table 1). Piglets were weaned at around 27 days of age (mean ± standard deviation (sd) weaning age: 27.4 ± 0.7 days of age) and relocated from the farrowing pen to the on-site weaning unit, where they remained for six weeks. After weaning, the same diet was provided across rounds. Further details of feed ingredients cannot be reported due to simultaneously performed studies by the farming facility and potential conflicts of interests.

**Table 1:** Diets and social mixing across rounds

*Replicated round*	*1*		*2*		*3*
*Days of age pre-weaning*	4–21	22–27	4–21	8–27	8–27
*Creep-feed pre-weaning*	Small pelleted feed A	Large pelleted feed A	Milk replacer	Small pelleted feed B	Small pelleted feed B
*Diet post-weaning from days of age 27–41*	Large pelleted feed A		Large pelleted feed A		Large pelleted feed A
*Social mixing at weaning*	None of the pens mixed		All four pens mixed		2 pens mixed, 2 pens unmixed

Dietary supplements during suckling phase in farrowing department, feed provided during post-weaning phase and social mixing at weaning per replicated round

Pens were spatially spread out across the stable unit (two in the front, one in the middle and one in the back). Pen floors were partially slatted (1.0 × 1.45 m) and partially solid (0.8 × 1.45 m). All pens were enriched with a hanging chain with a plastic ball and a piece of wood and a daily provision of a handful of wood shavings. For the purpose of a simultaneously performed study [51], two of the four pens additionally contained a rope attached to the metal chain as enrichment. Each pen was fitted with one nipple drinker, providing water ad libitum from the municipal drinking water system, which was checked daily by the animal caretakers. One feeding station with two feeding places provided feed ad libitum*.* Piglets were fed a standard commercial pelleted feed during the first two weeks post-weaning during all three replicated rounds (see Table 1). For the first two days, lights were on for 24 h, followed by a light period from 08:00–17:00. During the first two weeks, mean ambient temperature, relative humidity, CO_2_ levels and NH_3_ concentrations were 27° Celsius, 68%, 2200 parts per million (ppm) and 25 ppm, respectively. The animals were managed according to standard procedures of the facility.

### Study design

**Fig. 1:**
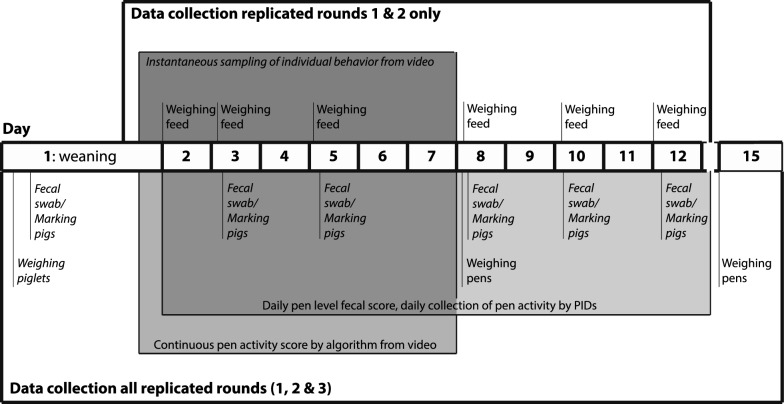
Timeline of data collection. Timeline per replicated round of data collected at the individual and pen level during only rounds 1 and 2 (above timeline) or during all rounds (1, 2 and 3; below timeline). Data collected on the individual level are shown in Italics, data collected on the pen level in regular typography. During replicated rounds 1 and 2, instantaneous sampling of individual behavior was performed from video during the first week post-weaning (day 1: 15:00–17:00, day 2–7: 08:00–17:00) and feed disappearance was measured periodically. In all three replicated rounds, pen activity was scored by an algorithm from video recordings from day 2 to 7 (09:00–17:00) and passive infrared detectors measured movement between day 2 and 12 (08:00–17:00). Fecal swabs were collected on days 1, 3, 5, 8, 10 and 12, when piglets where also marked individually. Pen scores were performed daily between day 2 and 12. In addition, piglets where weighed on days 1, 8 and 15. PIDs = passive infrared detectors

Data was collected during the first two weeks post-weaning during three replicated rounds between August and November 2021 (see Fig. 1 for a timeline of the study). A total of 72 piglets in 12 pens were included, with 24 piglets per round, six piglets per pen and four pens per round. Due to simultaneously performed studies by the facility, piglets were housed with their litter mates at weaning (all pens round 1; pen 1 and 4 in round 3) or socially mixed with unfamiliar conspecifics (all pens round 2; pen 2 and 3 round 3; see Table 1).

Only during the first two rounds (n = 48 piglets, n = 8 pens), behavior from individual piglets was analyzed from video recordings during the first week post-weaning by instantaneous scan sampling. Since it was not practically possible to measure the actual feed intake of the piglets and part of the feed was spilled through the grid floor, the term feed disappearance instead of feed intake is used. For the first two rounds, data on feed disappearance post-weaning were collected at the pen level three times weekly, by weighing the remaining feed in the early morning before video data was collected. Feed disappearance was calculated by adding up all provided feed per pen and subtracting the amount of left-over feed.

During all rounds, rectal swabs were collected from piglets six times post-weaning, on days 1, 3, 5, 8, 10 and 12. Fecal matter on the swabs was scored for color and consistency as a measure for PWD. Pen fecal color and consistency scores were evaluated daily between 2 and 12 days post-weaning by scoring stools present on the pen floor. Behavioral activity on the pen level was measured by an algorithm based on video recordings from 2 to 7 days post-weaning and by passive infrared detectors (PIDs) between 2 to 12 days post-weaning. Individual piglets were weighed at birth and at weaning. Pen level body weight was measured on days 8 and 15 post-weaning, and on day 8 they were weighed approximately 30–60 min before video recording for behavioral observations started.

### Data collection

#### Fecal scoring

- Individual level – swab fecal scores

To obtain a measure for the occurrence of PWD on the individual level, sterile rectal dry swabs (Sterile Dryswab™ Rayon, MWE, United Kingdom) were taken from individual piglets during all three replicated rounds (n = 12 pens, n = 72 piglets). Piglets were lifted and restrained by one experimenter, while a second experimenter inserted a swab ~ 3–5 cm into the rectum and gently moved it along the rectum wall for approximately ten seconds. The retrieved feces were rated directly on the swab for consistency and color by one of two experimenters, using an adaptation from the scoring system of Luise et al. [32] and Eriksen et al. [16] (see Table 2). We decided to score both color and consistency separately, as a summed score may biologically be difficult to interpret. Because it remains challenging to define when feces of a certain color and/or consistency can be classified as diarrhea, the terms aberrant stool or aberrant feces will be used instead when referring to our fecal scoring data. For color scoring, the color of the feces was compared to a print of reference colors. Yellow feces were only observed during the first few days (day 1 and day 3) post-weaning and were only observed to be of a solid consistency, likely related to their previous diet of maternal milk. Therefore, yellow feces were considered unrelated to PWD and scored as 0 for color. Green or white feces were never observed and therefore excluded from the scoring system. Inter-observer reliability for scoring ranged from sufficient (Cohen’s Kappa fecal color score: 0.68; p < 0.001, two observers) to good (Cohen’s Kappa fecal consistency score: 0.83; p < 0.001, two observers). Intra-observer reliability could not be tested as collected feces would dry rapidly, changing in both consistency and color. To allow for a binomial distribution for the data analysis, color and consistency stool scores of < 3 were considered as normal and scores of ≥ 3 as aberrant.

**Table 2:** Individual and pen level fecal scoring systems

*Score*	*Category*	*Consistency feces swab*	*Color feces swab*	*Consistency feces pen*	*Color feces pen*
*0*	Normal	Firm-solid	Dark brown yellow	Firm-solid	Dark brown or black
*1*	Normal	Firm-soft	Brown-grey	Firm-flat	Dark grey
*2*	Normal	Soft	Dark grey	Flat	Grey
*3*	Aberrant	Thin	Light grey	Flat-thin	Light grey
*4*	Aberrant	Watery-liquid	Light brown	Thin	Light brown
*5*	Aberrant	Liquid with blood and/or slime	Red /bloody Transparent	–	–

Scoring systems for scoring fecal consistency and color from rectal swabs of individual piglets [16, 32] and for scoring fecal matter on the pen floor (following the scoring system in use at the farming facility)

b. Pen level—pen floor fecal scores

Fecal scores on the pen level were conducted by four different animal caretakers during all three rounds (n = 12 pens) according to the scoring system already in use at the commercial farm and as shown in Table 2. Because of the grid floor, very watery feces (score 5) could easily be missed and was therefore not included in the scoring system. Inter-observer reliability could not be determined due to practical constraints, but pen level scores did show weak to moderate correlations to pooled fecal individual scores (see additional file AF1 Table 1). Color and consistency scores of < 3 were considered normal stools, while scores of ≥ 3 were considered as aberrant for the data analysis.

#### Behavioral activity

- Individual level—behavioral observations

For replicated rounds 1 and 2 (n = 8 pens, n = 48 piglets), individual piglet’s behaviors were scored from video footage. Above each pen, a camera (HIKVision, Hangzhou, China; Type DS-2CE16H5T-ITE 2.8 mm) was installed and videos were recorded during light hours. Videos were collected and stored continuously on a recorder (HIKVision, Hangzhou, China; Type DS-7204HUHI-K1/P). After the rectal swab was evaluated, the piglets were marked with an animal marker spray (MS spray marker, Schippers Bladel BV, Hapert, the Netherlands) to enable individual recognition on video recordings. Mutually exclusive behaviors as defined in Table 3 were scored by instantaneous sampling with a five minute interval from video. Recordings between 15:00–17:00 on the day of weaning (because weaning occurred between 08:00–13:00) and between 08:00–17:00 during 2 to 7 days post-weaning were scored using Observer XT software (version 15.0; Noldus Information Technology bv, Wageningen, The Netherlands), excluding times from 10 min before to 10 min after piglets were swabbed and marked (1 h 45 min between 09:20–12:30 of day 3 and 5). The observers were blind for the fecal scores during behavioral observations. Inter- and intra-observer reliability of three observers ranged from sufficient to almost perfect (see additional file AF2 Table 2).

**Table 3:** Ethogram for scoring individual behaviors

*Behavior*	*Description*	*Reference(s)*
*Lying*	Lying either on the side (recumbent) or on the belly (sternum). Piglet’s weight is not supported by legs, at least 3 legs are extended on the side/ “crossed” under the piglet. Piglet can be awake or asleep, with or without movements of head or parts of the body. Can be lying motionless or be performing other behaviors (such as social or exploratory behaviors) simultaneously, but is not eating or drinking at the feeder or drinker	[11]
*Standing*	Standing upright on 4 legs in one location without moving legs directionally, possible movements of head/body parts and minor movements of legs. Can be standing motionless or be performing other behaviors (such as social or exploratory behaviors) simultaneously, but is not eating or drinking at the feeder or drinker	[13]
*Walking*	Activity of all 4 legs resulting in directional movement/change in location. Can be performing other behaviors (such as social or exploratory behaviors) simultaneously	
*Feeding*	Head in feeder and/or chewing food in close proximity of feeder, with head directed towards feeder. Absence of performance of other behaviors and activity, remaining in one location (except standing or lying, which is not counted)	[5, 11, 13]
*Drinking*	Head in drinker/snout in contact with nipple waterer (if visible). Absence of performance of other behaviors, remaining in one location. (except standing or lying, which is not counted)	
*Other*	Simple activity behaviors that cannot be classified as any of the above (e.g. sitting)	

Ethogram used for scoring mutually exclusive individual activity behaviors from video recordings during the first week post-weaning

b. Pen level—automated algorithm

An automated algorithm developed and validated by an external partner (Serket BV, Amsterdam, the Netherlands; see Tangirala et al. [49]) was used to automatically detect pigs inside the pen and classify drinking, feeding, moving or inactivity (see additional file AF 3 video 1). The analysis was done at 10 frames per second (fps) of the recorded video data of all three rounds (n = 12 pens in total). The algorithm consisted of three major modules: (1) keypoint detection, (2) tracking for inter-frame keypoint association and (3) a consecutive behavior classification.

In total, 18 keypoints were used by the algorithm to detect individual pigs. The detections were fed to a tracker that associated the consecutive detections to trackstate based on average euclidean (i.e. shortest) distance per keypoint. The estimated current state of the tracks was the previous keypoint matched. After the tracker association, the active tracks (matched with detection on the frame) were fed to a behavior classification module. Each instance was investigated separately and classified as drinking, feeding, moving activity or inactivity (defined as when no other behavior was predicted). Behaviors were mutually exclusive, hierarchical (in the order as was written) and the inference executed in a lazy manner (inference stopped as the first action turns out positive). Feeding and drinking activity was predicted frame wise, based on the pigs nose-position in relation to the feeder and drinker masks, respectively (see additional file AF3 video 1). For feeding and drinking activity, a video of 30 min was manually annotated and resulted in a concordance correlation coefficient of 0.95 for feeding (performed in Rstudio [45], ρ: 0.95,95% confidence interval (CI): 0.95–0.96; Cb > 0.99) and 0.73 for drinking activity (ρ: 0.73; 95% CI: 0.73–0.75; Cb = 0.92). For detection of movement, only the torso-keypoints (excluding head) were used for distance calculations to reduce error, and they were smoothed with a 5 frame rolling window averaging. Threshold values for movement were based on dislocation values that were compared to manually annotated example videos. Performance was manually checked on a five-minute long video, resulting in a concordance correlation coefficient of 0.65 for the movement detector (ρ: 0.65; 95% CI: 0.59–0.71; Cb = 0.92). Lastly, all instances that had negative predictions from all of the behaviors, classifiers were considered inactive and missing detections were not assigned activity. Finally, the frame-wise data was merged to one second intervals and finally summed per day.

No distinction could be made between explorative behavior near the feeder or drinker (e.g. chewing or nosing part of the feeder/drinker) or actual feeding and drinking behavior. Therefore, the terms activity near the feeder or drinker are used. Due to spatial limitations, no more than three pigs could be feeding (based on video observations of the feeder with two feeding places) and no more than one pig could be drinking at the same time. Where the predicted values were higher than this, the numbers were clipped back and the remaining pigs were assigned to be inactive. Before comparison, all measurement points before 15:00 and after 17:00 for day 1 (approximately 2 h after weaning), before 09:00 (approximately 30–60 min after pens or feed were weighed) and after 17:00 for days 2–7 and for the time intervals when rectal swabs were taken (approximately 10 min before and after taking swabs and marking pigs, 1 h 45 min total) were discarded.

#### Passive infrared detectors (pen level only)

Passive infrared detectors (PIDs; Technical Development Studio, Wageningen University and Research, the Netherlands) equipped with a Panasonic EKMB1301112K motion sensor measured the motion of body heat in volts (V) during all three rounds (n = 12), recorded on a standard Lascar EL-USB-3 single-channel data logger (Lascar Electronics Inc., USA; EasyLog EL-USB-3; for more details see [51]). Each sensor was installed ~ 270 cm above each pen and was fitted with a short plastic tube (~ 20 cm) over the lens to ensure only movements inside the pen were measured. The PIDs had a maximum output of 3.3 V and measured the movement in each pen every 10 s between 08:00–17:00 from day 2 to 12 post-weaning. Data was extracted using EasyLog software (Lascar Electronics Inc., USA,EasyLog USB software version 7.7). To validate whether the PIDs could be used reliably to measure animal movement, the activity (active/moving or inactive/not moving) of piglets in two pens was scored manually every two minutes for 24 h during the first week post weaning. From this, the percentage of active piglets every two minutes was calculated and related to PID voltage output, which related strongly (Spearman correlation, ρ = 0.79; p < 0.0001; Veldkamp et al., in preparation), indicating that PIDs may be useful as valid detectors of piglet movement.

### Data analysis

All statistical analyses were performed in R statistical software [43] and RStudio [45]. Before analysis, all datasets were explored according to the steps of Zuur et al. [56]. Statistical significance was either determined by a p-value below the alpha threshold of 0.05 or when the 95% confidence interval (95%CI) of calculated odds ratio’s (ORs) did not include the value of 1. For determining inter- and intra-observer reliabilities of behavioral observations and fecal scoring (categorical data), Cohen’s kappa was calculated (package ‘irr’, [20]. For validating eating, drinking and moving activity as measured by the algorithm (continuous data, concordance correlation coefficients were calculated (packages ‘EpiR’ and ‘DescTools’ [46, 48],), which are less dependent on assumptions of normality [30]. Because of pertaining issues with singularity, glmmTMB models were used (package ‘glmmTMB’, [6] instead of generalized linear mixed effect models. In all analyses, separate models were run for fecal consistency and fecal color. For all analyses on the pen level, a random intercept for pen was applied as random term, while on the individual level, individual pigs were included as the random term. In all models, replicated round was incorporated as a fixed effect as there were insufficient levels to be included as a random term. This term accounts for all variation related to differences between rounds, including diets pre-weaning, social mixing at weaning, etc. Either day of fecal swab scoring was included, or day of pen floor fecal scoring was summarized in periods depending on the model (period 1: day 4–5 and period 2: day 6–8 in activity algorithm model,period 1: day 3–5, period 2: day 6–8 and period 3: day 9–12 in the PID model and feed disappearance models). The best model fit was acquired by backward stepwise regression using the drop1() function (package ‘lme4’, [2]) based on Akaike’s information criterion (AIC [9],) and fixed effects were dropped when resulting in a lower AIC. Residuals, outliers, dispersion and deviations from the distribution were tested by the package ‘DHARMa’ [22]. When fixed terms were effective predictors in the final model, the OR and associated 95%CI were calculated.

#### Individual behaviors—individual swab fecal scores

To determine whether behaviors performed on preceding days related to fecal swab scores for color and consistency, only the first occurrence of aberrant feces (based on either the color or consistency) was included. Subsequent scores were excluded from the dataset, because the behaviors preceding these scores could also be related to the existing aberrant fecal score. Two individuals showed aberrant feces from day one and were therefore completely excluded from the analysis. The occurrence of aberrant feces peaked around day 5, and even though many piglets still showed aberrant feces on day 8, no (for fecal color) or only few (for fecal consistency) new cases of aberrant feces occurred after day 5. Therefore, only fecal scores of days 3 and 5 had sufficient variation to be included in the models as the dependent variable.

The total frequency of the behaviors were summed for each day of scoring and the proportion of each behavior compared to all behaviors was calculated to correct for the fewer hours observed on the day of weaning and on days of taking rectal swabs. These proportions resulted in very wide and unprecise 95% confidence intervals and therefore, the behaviors were categorized in low and high proportions. When behaviors occurred less than the mean of the behavior occurring across all piglets, days and rounds (overall mean), it was considered a low proportion, while if it occurred equal to or higher it was considered a high proportion. The binomial variable for each behavior on the day of scoring, and one and two days before fecal scoring were included as fixed effects. Drinking behavior was not observed enough to be included in the analyses, while lying behavior was negatively collinear to walking and standing (see additional file AF4 Fig. 1) and was therefore excluded from the analyses.

#### Pen activity algorithm—pen floor fecal scores

**Fig. 2:**
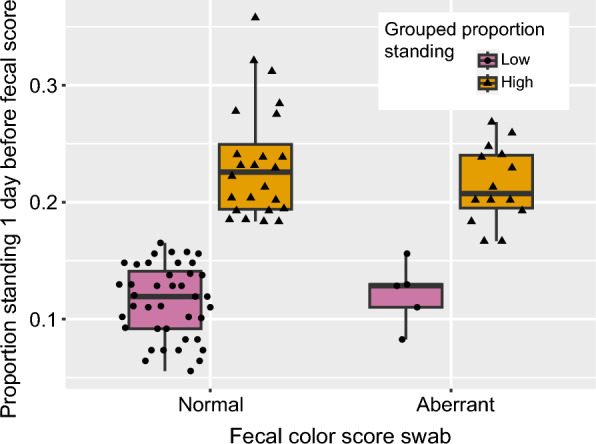
Proportion standing one day before fecal scoring from swabs in relation to fecal color. Instances (individual points, n = 46 piglets scored once or twice) where individual pigs were given a normal or aberrant fecal color score in relation to the proportion standing one day before performing the fecal score, grouped in low proportion (values lower than the overall mean) or high proportion (values equal to or higher than the overall mean). Fecal scores performed on day 3 and 5 are included, with only including data of the first instance of aberrant fecal color score per pig. Boxplot horizontal lines indicate medians, boxes indicate interquartile range and vertical lines indicate 1.5 times the interquartile range

The algorithm output was the number of pigs performing each behavior per second (drinking, feeding, moving or inactivity), summed per day. These outputs were normalized, representing the percentage of the total data an average pig per pen was performing the said activity for each analyzed day. On day 1, total activity near the feeder and drinker were very high compared to the subsequent days, despite being based on only two instead of eight observation hours, and data were therefore excluded from the analysis. These high values are likely caused by high levels of exploration of both the feeder and drinker, as observed from videos, as piglets were moved to their new pens only a few hours prior. Because inactivity one and two days before fecal scoring was negatively collinear to all other behaviors, it was excluded from the analyses (see additional file AF5 Fig. 2). Similarly, as moving activity one and two days before fecal scoring and feeding and drinking activities were collinear, only the ones resulting in the lowest AIC were kept in the model (i.e. either moving one day before or moving two days before fecal scoring and either drinking activity or feeding activity one or two days before fecal scoring).

Pen fecal scores were performed from day 2 until day 12 on the pen level, while the algorithm was used to score video recordings from day 2 to 7. Because previously scored aberrant feces may result from different individuals, it was not possible to exclude subsequent scores from the dataset. Therefore, all pen fecal scores from day 4 to 8 and the behavioral activities occurring one and two days before the fecal score were included in the models.

#### Additional exploratory analyses

To explore whether a simple measure of pen activity could be related to fecal pen scores, the output rescaled to kilovolts of the passive infrared detectors (PIDs) one and two days before and on the day of pen fecal scoring were included as predictors in the models. Because these PID outputs were highly correlated (ranging from ρ = 0.80–0.91), only the one resulting in the lowest AIC was included in the final model. In addition, it was analyzed whether the feed disappearance per day on the pen level in rounds 1 and 2 affected pen fecal scoring (n = 8). Lastly, it was explored whether birth or weaning weight affected the odds of developing aberrant feces post-weaning. A general linear model (package ‘glm2’, [37]) was run on the individual piglet level (n = 72) with developing aberrant feces (yes/no) as the dependent variable and birth- and weaning weight as independent variables.

## Results

### Descriptive results

For descriptive results on body weight, see additional file AF6 Table 3. For descriptive results on behavior and fecal scores on the individual and pen level, see additional file AF7 Table 4.

- Individual level

On the individual level, piglets showed the highest proportion of lying behavior (round 1: 0.76 ± 0.05; round 2: 0.67 ± 0.06), followed by standing (round 1: 0.16 ± 0.04; round 2: 0.19 ± 0.05), eating (round 1: 0.05 ± 0.02; round 2: 0.07 ± 0.02) and walking (round 1: 0.02 ± 0.01; round 2: 0.04 ± 0.01). Aberrant fecal color scores mostly occurred on day 3 (round 1: n = 3; round 2: n = 6) and 5 (round 1: n = 8; round 2: n = 5), while aberrant fecal consistency scores occurred mostly on day 5 (round 1: n = 12; round 2: n = 14). On day 3 or 5, at least 1 piglet per pen had an aberrant fecal color score and on day 5, at least 2 piglets per pen showed an aberrant fecal consistency.

b. Pen level

At the pen level, piglets showed highest percentages of inactivity (round 1: 92.1 ± 3.57%; round 2: 89.5 ± 1.86%; round 3: 88.0 ± 1.98%), followed by activity at the feeder (round 1: 5.19 ± 2.48%; round 2: 6.71 ± 2.51%; round 3: 8.39 ± 1.61%), moving activity (round 1: 1.47 ± 0.82%; round 2: 2.40 ± %0.90; round 3: 2.18 ± 1.02%) and activity at the drinker (round 1: 1.25 ± 1.46%; round 2: 1.39 ± 0.98%; round 3: 1.45 ± 1.18%). Most fecal pen color and consistency scores were aberrant during days 6–8 of fecal scoring (period 2 in analysis) (fecal pen color score: round 1: n = 1; round 2: n = 9; round 3: n = 10; fecal pen consistency score: round 1: n = 5; round 2: n = 11; round 3: n = 12). During period 2, all pens across all rounds except 3 (n = 9) showed an aberrant fecal color on the pen level and all pens except 1 (n = 11) had an aberrant fecal pen consistency score at least once.

### Individual behaviors—fecal swab scores (individual level)

- Fecal swab color model

Individual piglets that proportionally showed higher levels of standing as measured by instantaneous sampling one day before fecal scoring had a 4.79 times higher odds of an aberrant fecal color score than pigs that stood less one day before fecal scoring (high standing (normal fecal color: n = 24; aberrant fecal color n = 14) vs low standing (normal fecal color: n = 40; aberrant fecal color: n = 5); OR: 4.79; 95%CI: 1.51–15.3) (see Fig. 2 and Table 4A). Day of fecal scoring, pen or round did not affect the odds of an aberrant fecal color score.

**Table 4:** Results of main and exploratory analyses

**A**	Individual behaviors–fecal swab scores							Pen activity algorithm–fecal pen scores						
Response	Fixed effect	Sample size (piglets)	Obs	Comparison (normal (N) vs. Aberrant (A))	Odds ratio	2.5% Lower limit	97.5% Upper limit	Fixed effect	Sample size (pens)	Obs	Comparison (normal (N) vs. aberrant (A))	Odds ratio	2.5% Lower limit	97.5% Upper limit
*Fecal color score*	Proportion standing Min1 binary (high (H) vs. low (L))	46	83	HN: 24 HA: 14 LN:40 LA: 5	**4.79**	**1.51**	**15.3**	%Moving activity Min2	12	60	1.43 ± 0.58% vs. 2.18 ± 0.74%	**6.14**	**1.26**	**29.8**
								%Drinking activity Min1	12	60	0.99 ± 0.44% vs. 0.96 ± 0.22%	0.06	0.003	1.39
	Proportion walking Min0 binary (high (H) vs. low (L))	46	83	HN: 22 HA: 3 LN: 42 LA: 16	0.34	0.09	1.36	Round (2 vs. 1)	12	60	2N: 4 1N:19 2A: 16 1A: 1	**51.1**	**4.05**	**645**
								Round (3 vs. 1)	12	60	3N: 9 1N:19 3A: 11 1A: 1	**28.5**	**2.06**	**395**
*Fecal consistency score*	Proportion walking Min2 binary (high (H) vs. low (L))	44	85	HN: 19 HA: 9 LN: 40 LA: 17	2.42	0.66	8.88	%Moving activity Min2	12	60	1.25 ± 0.63% vs. 2.04 ± 0.67%	**4.77**	**1.11**	**20.6**
								%Eating activity Min1	12	60	6.00 ± 2.23% vs. 8.24 ± 1.63%	1.59	0.90	2.80
	Day (day 5 (d5) vs. day 3 (d3))	44	85	d5N: 18 d5A: 23 d3N: 41 d3A: 3	**22.3**	**5.34**	**92.9**	Round (2 vs. 1)	12	60	2N: 1 1N:14 2A: 19 1A: 6	**15.9**	**1.46**	**173**
								Round (3 vs. 1)	12	60	3N: 6 1N: 14 3A: 14 1A: 6	1.30	0.20	8.63

**B**	Pen activity passive infrared detectors–fecal pen scores							Performance–fecal swab/pen scores						
Response	Fixed effect	Sample size (pens)	Obs	Comparison (normal (N) vs. Aberrant (A))	Odds ratio	2.5% Lower limit	97.5% Upper limit	Fixed effect	Sample size	Obs	Comparison (normal (N) vs. Aberrant (A))	Odds ratio	2.5% Lower limit	97.5% Upper limit
*Fecal color score*	PID Min1 (kVolt)	12	97	4.97 ± 1.51 vs. 4.84 ± 1.90	0.83	0.57	1.19	Birth weight (kg)	72 (piglet)	72	1.62 ± 0.36 vs. 1.57 ± 0.30	0.999	0.998	1.00
	Period (2 (p2) vs. 1 (p1))	12	97	p2N: 16 p2A: 14 p1N: 21 p1A: 7	**5.89**	**1.42**	**24.4**	Weaning weight (kg)	72 (piglet)	72	8.66 ± 0.93 vs. 8.65 ± 1.07	1.00	0.999	1.00
	Period (3 (p3) vs. 1 (p1))	12	97	p3N: 33 p3A: 6 p1N: 21 p1A: 7	0.78	0.18	3.38	Period (all)	8 (pen)	49	p1N: 13 p1A: 5 p2N: 8 p2A: 8 p3N: 14 p3A: 1	0.67	0.21	2.19
	Round (2 vs. 1)	12	97	2N: 19 1N: 33 2A: 19 1A: 1	**70.9**	**7.24**	**695**	Round (2 vs. 1)	8 (pen)	49	2N: 13 1N: 22 2A: 13 1A: 1	**21.6**	**2.26**	**208**
	Round (3 vs. 1)	12	97	3N: 18 1N: 33 3A: 7 1A: 1	**18.5**	**1.82**	**188**	Daily feed disappearance (kg)	8 (pen)	49	1.69 ± 0.96 vs. 1.77 ± 0.70	0.97	0.28	3.37
*Fecal consistency score*	PID Min1 (kVolt)	12	97	5.20 ± 1.51 vs. 4.49 ± 1.71	0.66	0.42	1.05	Birth weight	72 (piglet)	72	1.58 ± 0.30 vs. 1.60 ± 0.35	1.00	0.999	1.00
								Weaning weight	72 (piglet)	72	8.67 ± 0.86 vs. 8.65 ± 1.07	1.00	0.999	1.00
	Period (2 vs. 1)	12	97	p2N: 7 p2A: 23 p1N: 18 p1A: 10	**16.3**	**2.89**	**92.1**	Period (all)	8 (pen)	49	p1N: 14 p1A: 4 p2N: 5 p2A: 11 p3N: 15 p3A: 0	1.36	0.42	4.43
	Period (3 vs. 1)	12	97	p3N: 36 p3A: 3 p1N: 18 p1A: 10	0.21	0.04	1.09	Round (2 vs. 1)	8 (pen)	49	2N: 14 1N: 20 2A: 12 1A: 3	**12.1**	**1.92**	**76.6**
	Round (2 vs. 1)	12	97	2N: 18 1N: 28 2A: 20 1A: 6	**38.0**	**5.09**	**284**	Daily feed disappearance (kg)	8 (pen)	49	1.80 ± 0.96 vs. 1.50 ± 0.69	0.32	0.09	1.16
	Round (3 vs. 1)	12	97	3N: 16 1N: 28 3A: 9 1A: 6	5.61	0.88	35.9							

(**A**) Results of main analyses on individual behavior and fecal scores from swabs and pen activity as measured by the algorithm and fecal pen scores and (**B**) results of exploratory analyses on voltage measured by the passive infrared detectors (PIDs) and fecal pen scores (period 1: day 3–5; period 2: day 6–8; period 3: day 9–12) and production parameters and fecal swab/pen scores. Bold results indicate significance (95% confidence interval does not include 1). Min0 = on day of fecal scoring; Min1 = one day before fecal scoring; Min2 = two days before fecal scoring. Obs. = number of observations in model. kVolt = kilovolt, kg = kilograms. Comparison column indicates either number of observations in each category of the predictor or mean ± sd of the predictor for both normal or aberrant fecal scores

b. Fecal swab consistency model

No relation between behavior as measured by instantaneous sampling and aberrant fecal consistency was found, but on day 5 the odds to observe aberrant fecal consistency were 22.3 times higher than on day 3 (day 5 (normal fecal consistency: n = 18; aberrant fecal consistency n = 23) vs day 3 (normal fecal consistency: n = 41; aberrant fecal consistency: n = 3); OR: 22.3; 95%CI: 5.34–92.9) (see Table 4A). The odds of aberrant fecal consistency were not affected by pen or round.

### Pen activity algorithm—fecal pen scores (group level)

- Fecal pen color model

**Fig. 3:**
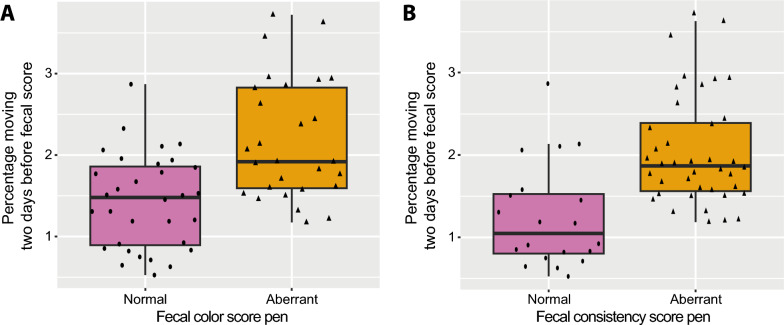
Percentage of moving two days before fecal pen scoring in relation to color and consistency. The percentage of moving activity as measured by the algorithm two days before performing the fecal score in relation to (**A**) instances (individual points, n = 12 pens) where pens were scored to contain a normal or aberrant fecal color and (**B**) instances (individual points, n = 12 pens) where pens were scored to contain a normal or aberrant fecal consistency. Fecal scores of day 4–8 are included. Boxplot horizontal lines indicate medians, boxes indicate interquartile range and vertical lines indicate 1.5 times the interquartile range

Odds of an aberrant fecal color score were 6.14 times higher when the moving activity two days before scoring, as measured by the algorithm, increased by 1% (number of pigs moving per second summed per day, percentage of moving activity compared to total behavioral activities) (aberrant (2.18 ± 0.74%) vs. normal colored feces (1.43 ± 0.58%); OR: 6.14; 95%CI: 1.26–29.8; see Table 4A and Fig. 3A). In other words, when pens showed a higher moving activity two days before pen fecal scoring compared to pens that showed less moving activity, the odds of an aberrant fecal color score increased. Finally, there was an effect of replicated round, with odds of an aberrant fecal color score being 51.1 times higher in round 2 and 28.5 times higher in round 3 compared to round 1 (round 2 (normal fecal color: n = 4; aberrant fecal color: n = 16) and round 3 (normal fecal color: n = 9; aberrant fecal color: n = 11) vs. round 1 (normal fecal color: n = 19; aberrant fecal color: n = 1); OR: 51.1; 95%CI: 4.05–645 and OR: 28.5; 95%CI: 2.06–395; see Table 4A).

b. Fecal pen consistency model

Odds of aberrant fecal consistency at the pen level were 4.77 times higher when the moving activity as measured by the algorithm two days before fecal scoring increased by 1% (aberrant (1.25 ± 0.63%) vs. normal consistency feces (2.04 ± 0.67%); OR: 4.77; 95%CI: 1.11–20.6; see Table 4A and Fig. 3B). Thus, when pens showed a higher moving activity compared to pens that moved less two days before pen fecal scoring, the odds of an aberrant fecal consistency score increased. Moreover, odds of aberrant fecal consistency were 15.9 times higher in replicated round 2 compared to 1 (round 2 (normal fecal consistency: n = 1; aberrant fecal consistency: n = 19) vs. round 1 (normal fecal consistency: n = 14; aberrant fecal consistency: n = 6); OR: 15.9; 95%CI: 1.46–174; see Table 4A).

### Additional exploratory analyses

#### Pen activity passive infrared detectors (PIDs)—fecal pen scores (group level)

- Fecal pen color model

Kilovoltage measured by the PIDs one or two days before fecal scoring or on the day of scoring did not affect the odds for detection of an aberrant fecal color score at the pen level. However, in replicated rounds 2 and 3, odds of an aberrant fecal color score were 70.9 and 18.5 times higher than in round 1, respectively (round 2 (normal fecal color: n = 19; aberrant fecal color: n = 19) and round 3 (normal fecal color: n = 18; aberrant fecal color: n = 7) vs. round 1 (normal fecal color: n = 33; aberrant fecal color: n = 1); OR: 70.9; 95%CI: 7.24–695 and OR: 18.5; 95%CI: 1.82–188) (see Table 4B). Odds of an aberrant fecal color score were 5.89 times higher in period 2 (day 6–8) compared to 1 (day 3–5) (period 2 (normal fecal color: n = 16; aberrant fecal color: n = 14) vs. period 1 (normal fecal color: n = 21; aberrant fecal color: n = 7); OR: 5.89; 95%CI: 1.42–24.4) (see Table 4B).

b. Fecal pen consistency model

Similar to the odds of an aberrant pen fecal color score, the odds of an aberrant pen fecal consistency score was not affected by the kilovoltage measured by the PIDs one or two days before fecal scoring or on the day of scoring. In round 2, odds of an aberrant fecal consistency score were 38.0 times higher than in round 1 (round 2 (normal fecal consistency: n = 18; aberrant fecal consistency: n = 20) vs. round 1 (normal fecal consistency: n = 28; aberrant fecal consistency: n = 6); OR: 38.0; 95%CI: 5.09–284) (see Table 4B). Odds of an aberrant fecal consistency score were 16.3 times higher in period 2 (day 6–8) compared to 1 (day 3–5) (period 2 (normal fecal consistency: n = 7; aberrant fecal consistency: n = 23) vs. 1 (normal fecal consistency: n = 18; aberrant fecal consistency: n = 10); OR: 16.3; 95%CI: 2.89–92.1) (see Table 4B).

#### Performance parameters—individual fecal swab and pen fecal scores

On the individual level, no effect of birth- or weaning weight was found on developing feces of an aberrant fecal color or consistency post-weaning. Mean daily feed disappearance in replicated rounds 1 and 2 did not affect the odds of an aberrant fecal color or consistency score on the pen level. However, in round 2, odds were 21.6 higher of an aberrant fecal color score compared to round 1 (round 2 (normal fecal color: n = 13; aberrant fecal color: n = 13) vs. round 1 (normal fecal color: n = 22; aberrant fecal color: n = 1); OR: 21.6; 95%CI: 2.26–208). For fecal consistency, odds were 12.1 times higher in round 2 compared to round 1 (round 2 (normal fecal consistency: n = 14; aberrant fecal color: n = 12) vs. round 1 (normal fecal consistency: n = 20; aberrant fecal consistency: n = 3); OR: 12.1; 95%CI: 1.92–76.6) (see Table 4B). In both models for feed disappearance (fecal pen color and consistency), period of scoring (period 1: day 3–5, period 2: day 6–8, period 3: day 9–12) did not affect the odds of an aberrant fecal score (see Table 4B).

## Discussion

The aim of this study was to establish whether individual- or pen level changes in behavior can be used as early indicators of aberrant feces in weaned piglets. Video recordings of behaviors were scored manually using instantaneous sampling at the individual level, while an existing algorithm was used to score behavioral activity continuously on the pen level. Aberrant feces were scored either from swabs on the individual level or on the pen floor at the group level. Aberrant fecal scores peaked around 5 to 8 days after weaning, which corresponds with earlier findings [34].

We expected that lethargy associated with the onset of disease might lead to inactivity, but contrary to our expectations, we found that individual piglets had higher odds of an aberrant fecal color score when they showed higher levels of standing on the previous day compared to piglets that stood less. These results are in line with recent findings [28] on the group level, where piglets in pens untreated with antimicrobials and having looser feces showed a higher proportion of standing postures during the first six days post-weaning. It was argued that this increase in standing could be caused by increased defecation or drinking behavior, but in our study, standing was not scored when piglets were drinking (scored as drinking instead) or defecating (scored as other instead). Furthermore, increased standing has likewise been observed in pigs infected by *Salmonella* [44], suggesting that the infected pigs might be restless in response to the infection.

On the pen level, standing behavior was not measured by the algorithm, but instead it was found that pens showing higher moving activity two days before had increased odds of getting an aberrant fecal color or consistency score. Walking behavior scored manually on the individual level (directional movement with all four legs) and moving activity as scored by the algorithm (movement of entire, front or back part of torso) were differentially defined, possibly explaining why this effect was not found for walking behavior on the individual level. Nevertheless, together these findings indicate that an increase in standing or moving activity may be important early indicators of PWD, possibly indicating some type of restlessness before the onset of clinical signs, perhaps caused by abdominal pains. Alternatively, vulnerable piglets may initially spend more time inactive in response to the weaning process and after a period of having fasted, show more active behaviors including standing and explorative behavior due to hunger. This increase in standing or moving activity may therefore be detectable right before the onset of diarrhea and could be an important early indicator of PWD. In contrast, previous studies indicate that once an animal is ill, it shows an increase in lying behaviors or an overall lethargy [21], which has also been found for piglets suffering from PWD [31]. However, instead of focusing on behavioral changes once the illness has been established, our aim was to investigate whether behavioral changes occur before the onset of aberrant feces. Therefore, we found evidence that individual or groups of piglets may first show an increase in standing or moving activity before showing signs of diarrhea.

In our exploratory analyses of relating a simple measure of overall pen activity as measured by passive infrared detectors (PIDs), to aberrant fecal consistency or color, no effect was found. The difference in method may explain why moving activity was found to be an early indicator of PWD when measured with the algorithm but not when using PIDs. PID activity also includes movements related to drinking and feeding, while in the analyses of both the individual and pen level behaviors, neither drinking nor feeding activity affected the odds of aberrant feces. In addition, increased standing does not result in a voltage change, while on the individual level standing was the only behavior that was higher in piglets developing PWD. On the group level, both subtle and larger changes in posture as scored by the algorithm are weighed equally, as only the total number of pigs moving was scored. In contrast, the PIDs measure all movement in the pen in different degrees of voltage, depending on how much and how energetic movement is taking place. Therefore, our results implicate that PID activity is not sufficiently accurate to be used as an early indicator of PWD. However, it should be noted that overall, the piglets showed relatively high levels of inactivity, independent of fecal color or consistency on both the individual and group level. This could be due to the relatively small pen size used in this study, as only little space in absolute terms was available to perform more active behaviors, despite the relative space per piglet being within the range of conventional conditions and compliant with legal requirements. Activity measured by PIDs as an early indicator of PWD should therefore be researched under different conditions with more opportunity to show active behaviors.

Contrary to our predictions, both drinking and feeding activity were not effective early indicators of PWD, despite the different diets provided pre-weaning across rounds. For feeding activity, this is in line with recent findings, while drinking has been reported to increase in relation to PWD [28, 35]. The number of visits or duration spent at the feeder or drinker may in fact not be representative for the actual feed and water intake [7]. However, in our study and in line with Engelsmann et al. [15], a decrease in feed intake in relation to an increased risk of PWD as repeatedly reported [14, 24, 25, 50] was not found. It has been argued that the lower feed intake is initially caused by the overall stress experienced immediately post-weaning and the neophobia of novel feed [8, 27, 36]. Alternatively, once signs of PWD can be detected, diarrhea-affected pigs may simply eat less, meaning that feed intake would only decline once diarrhea can already be observed. Similarly, piglets may increase their water intake only after losing substantial water through loose feces, as they may first deplete their physical fluid reserves before replenishing this deficit. A recent study actually did not find a difference in water intake between healthy and affected piglets before the onset of signs or even on the first day of showing loose feces [50].

The absence of an effect of feeding and drinking behavior in our study may also be due to methodological constraints. On the individual level, the five-minute interval of the instantaneous sampling method, may have resulted in a substantial amount of feeding events being missed and even insufficient data on drinking events. Furthermore, the categorization of behaviors in high and low proportions due to statistical issues may have masked any differences in eating as proportions were already very low. On the pen level, activity near the feeder and drinker on the group level also included explorative behaviors and because of the small pens possibly even proximity at the feeder or drinker. Feed disappearance was only measured at the pen level every few days and with a small sample size (n = 8). Measuring actual feed or water intake at the individual level over time may be more reliable and predictive indicators for the development of PWD [7, 15]. Here, also the group size and small pens may have affected the results, as piglets had relatively more feeding and drinking places per pig than in more conventional larger groups, providing clinically affected pigs relatively less competition and more opportunity to eat and drink.

On the pen level, we found an effect of round on the odds of an aberrant fecal color and consistency score, with the odds in round 2 and often 3 being higher than in round 1, depending on the model. Besides inherent differences between rounds such as climatic conditions, pre-weaning diets and social mixing at weaning differed across rounds (see Table 1). In round 1, piglets were supplemented with the same pelleted feed in the suckling phase as in the post-weaning phase, while they were not socially mixed at weaning. In round 2 piglets were supplemented with another pelleted feed in the farrowing stable compared to the nursery stable and all pens were socially mixed at weaning, which is an important risk factor of PWD [4, 40, 42, 54]. Finally, in round 3, the supplemented feed type differed in the suckling phase from the post-weaning phase, but only half of the pens were socially mixed. This may partially explain why the odds on aberrant fecal scores were highest in round 2, followed by round 3 and lowest in round 1.

In contrast to our expectations, we did not find an effect of round in our analyses on individual level behavior. One explanation could be that adjusted datasets were used in these analyses where only the first instances of aberrant feces per piglet were included, whereas on the group level all fecal scores were included. This may indicate that social mixing and pre-weaning diets may not only increase chances on the occurrence of aberrant feces or even PWD, but may also result in a longer duration of aberrant feces on the pen level, but this cannot be concluded from our study. In addition, when only few piglets per pen showed aberrant feces, this could be detected on the individual level, but may be missed on the pen level, resulting in different numbers of aberrant fecal scores per round when measured on the individual or pen level.

Although our aim was to establish early indicators of PWD by studying behaviors one and two days before the onset of clinical signs, on both the individual- and group level we cannot ascertain entirely that these behavioral changes occurred only before first showing signs of diarrhea. In the analysis on the individual level, fecal swabs were taken every other day, meaning that diarrhea could have occurred for the first time the day before. It was decided to take rectal swabs only every other day because swabbing can in turn influence behavior. For the analysis on the pen level using the algorithm data, we could not include only first time instances of aberrant feces, as these could be instances of different pigs in the same pen. However, these scores could also be from the same pigs and therefore behavioral changes related to these scores may not occur before the onset of PWD but when aberrant feces were already detectable.

Nevertheless, more standing of individual pigs and higher levels of moving activity at the group level may be important to monitor as early indicators of PWD. As the etiology of PWD is complex and related to the specific stress response and immune competence of piglets, focusing on individuals early can aid in more targeted interventions and less antimicrobial use on the pen or barn level. Additionally, further interventions reducing stress around weaning can aid in preventing other piglets to be affected. The development of algorithms to detect behavioral abnormalities on the individual level may therefore be valuable, not only for monitoring behavior in relation to PWD, but also for monitoring other disease and welfare issues. Using this or similar algorithms to detect behavioral changes on different farms, group sizes and ages can further reveal how behavior may function as an early indicator of illnesses in pigs. Similarly, further advancement in the field can result in more complex behaviors such as social interaction and exploration to be measured automatically in practice and to be studied in relation to impaired health. Moreover, although early detection can benefit disease control, changes in management (e.g. gradual and later weaning) and housing systems (e.g. early socialization during suckling) aimed at prevention should also always be considered [10, 18, 26].

## Conclusion

Our results indicate that higher levels of standing in piglets may be an important early indicator of post-weaning diarrhea one day before the onset of clinical signs. Furthermore, on the pen level, piglets may show increased moving activity before or when signs of diarrhea are apparent. A promising avenue towards improved and targeted health monitoring and treatment may be the expansion of algorithms that are currently able to detect behavioral abnormalities on group level towards detection on individual level.

## Supplementary Information

Additional file 1.

Additional file 2.

Additional file 3.

Additional file 4.

Additional file 5.

Additional file 6.

Additional file 7.

## Data Availability

The datasets used and/or analysed during the current study are available from the corresponding author on reasonable request.

## Abbreviations

95%CI: 95% Confidence interval
AIC: Akaike’s information criterium
fps: Frames per second
OR: Odds ratio
PIDs: Passive infrared detectors
ppm: Parts per million
PWD: Post-weaning diarrhea
sd: Standard deviation
V: Volts
