# Supplementary material for: Early behavioral indicators of aberrant feces in newly-weaned piglets
Source: Porcine Health Manag. 2024 Nov 5;10:47. doi: 10.1186/s40813-024-00396-4 (PMC11536707; doi:10.1186/s40813-024-00396-4)
Supplement: Supplementary file 1 — Additional file 1. [file 40813_2024_396_MOESM1_ESM.docx]

**AF1 Table 1. Correlations mean individual fecal scores per pen and pen level fecal scores.** Spearman rank correlations (*ρ*) for mean individual fecal color and consistency and pen color and consistency for n=71 scores conducted. **p<0.01, ***p<0.001.

| *Individual measure* | *Pen measure* | *ρ* | *S-value* |
| --- | --- | --- | --- |
| Mean individual color score per pen | Pen color score | 0.31** | 41155 |
| Mean individual consistency score per pen | Pen consistency score | 0.62*** | 22765 |
