# Supplementary material for: Early behavioral indicators of aberrant feces in newly-weaned piglets
Source: Porcine Health Manag. 2024 Nov 5;10:47. doi: 10.1186/s40813-024-00396-4 (PMC11536707; doi:10.1186/s40813-024-00396-4)
Supplement: Supplementary file 2 — Additional file 2. [file 40813_2024_396_MOESM2_ESM.docx]

**AF2 Table 2. Inter-observer reliability of behavioral observations**. Inter- and intra-observer reliability results for behavioral observations on the individual level. ***p<0.001.

|  | *Inter-observer* | *Intra-observer 1* | *Intra-observer 2* | *Intra-observer 3* |
| --- | --- | --- | --- | --- |
| *Total number of observations* | 444 | 438 | 438 | 420 |
| *Number of raters/*  *repetitions* | 3 | 2 | 2 | 2 |
| *Fleiss’ Kappa*  *All behaviors* | 0.82*** | 0.89*** | 0.86*** | 0.92*** |
| *z-statistic*  *All behaviors* | 64.4 | 25.4 | 26.1 | 26.6 |
| *Fleiss’ Kappa*  *Lying* | 0.91*** | 0.93*** | 0.94*** | 0.95*** |
| *z-statistc*  *Lying* | 60.0 | 19.5 | 19.6 | 19.5 |
| *Fleiss’ Kappa Standing* | 0.79*** | 0.86*** | 0.83*** | 0.90*** |
| *z-statistic*  *Standing* | 51.9 | 18.1 | 17.4 | 18.4 |
| *Fleiss’ Kappa Walking* | 0.60*** | 0.77*** | 0.66*** | 0.79*** |
| *z-statistic*  *Walking* | 39.7 | 16.1 | 13.8 | 16.2 |
| *Fleiss’ Kappa Feeding* | 0.90*** | 0.98*** | 0.90*** | 0.96*** |
| *z-statistic*  *Feeding* | 58.9 | 20.5 | 18.9 | 19.7 |
| *Fleiss’ Kappa Drinking* | 0.90*** | 1.00*** | 1.00*** | 1.00*** |
| *z-statistic*  *Drinking* | 59.1 | 20.9 | 20.9 | 20.5 |
