# Supplementary figures and images for: Early behavioral indicators of aberrant feces in newly-weaned piglets
Source: Porcine Health Manag. 2024 Nov 5;10:47. doi: 10.1186/s40813-024-00396-4 (PMC11536707; doi:10.1186/s40813-024-00396-4)

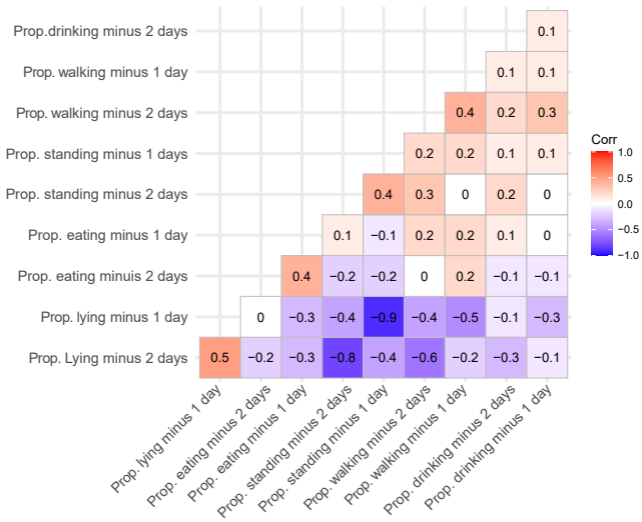

Supplement: Supplementary file 4 — Additional file 4. [file 40813_2024_396_MOESM4_ESM.pdf]

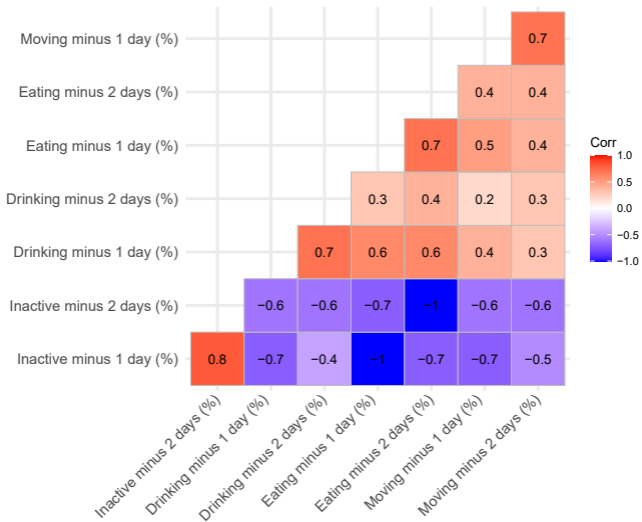

Supplement: Supplementary file 5 — Additional file 5. [file 40813_2024_396_MOESM5_ESM.pdf]
