# Supplementary material for: Early behavioral indicators of aberrant feces in newly-weaned piglets
Source: Porcine Health Manag. 2024 Nov 5;10:47. doi: 10.1186/s40813-024-00396-4 (PMC11536707; doi:10.1186/s40813-024-00396-4)
Supplement: Supplementary file 6 — Additional file 6. [file 40813_2024_396_MOESM6_ESM.docx]

**AF6 Table 3. Body weight data per pen and round.** Birth- and weaning weight (mean±sd) per pen per round collected at the individual level and subsequent mean body weights (kg) collected at pen level at 8 and 15 days post-weaning.

| *Round* | *Pen* | *Birth weight (kg) Mean±sd* | *Weaning weight (kg) Mean±sd* | *Mean weight (kg) day 8 post-weaning* | *Mean weight (kg) day 15 post-weaning* |
| --- | --- | --- | --- | --- | --- |
| *1* | *1* | 1.40±0.10 | 8.04±0.56 | 9.42 | 11.38 |
|  | *2* | 0.95±0.12 | 7.40±1.17 | 7.78 | 10.42 |
|  | *3* | 1.52±0.30 | 8.48±1.20 | 8.40 | 10.45 |
|  | *4* | 1.90±0.13 | 9.32±0.32 | 9.80 | 11.95 |
| *2* | *1* | 1.57±0.22 | 9.43±0.03 | - | 12.52 |
|  | *2* | 1.74±0.25 | 9.44±0.06 | 10.07 | 12.78 |
|  | *3* | 1.68±0.11 | 9.72±0.12 | 10.45 | 13.00 |
|  | *4* | 1.50±0.28 | 7.79±0.19 | 8.35 | 10.60 |
| *3* | *1* | 1.50±0.12 | 7.97±1.28 | 8.68 | 10.88 |
|  | *2* | 1.68±0.21 | 8.41±0.52 | 9.40 | 11.92 |
|  | *3* | 1.70±0.38 | 8.61±0.70 | 9.88 | 12.20 |
|  | *4* | 1.99±0.29 | 9.27±0.62 | 10.70 | 12.73 |
| *Total* | | 1.59±0.33 | 8.66±0.98 | 9.36±0.94 | 11.74±0.96 |
