# Supplementary material for: Early behavioral indicators of aberrant feces in newly-weaned piglets
Source: Porcine Health Manag. 2024 Nov 5;10:47. doi: 10.1186/s40813-024-00396-4 (PMC11536707; doi:10.1186/s40813-024-00396-4)
Supplement: Supplementary file 7 — Additional file 7. [file 40813_2024_396_MOESM7_ESM.docx]

**AF7 Table 4. Descriptive results of behavioral and fecal scoring data on individual- and pen level.** (**A**) Proportions of behaviors and number of fecal swab scores scored on the individual level and (**B**) percentages of behavioral activity and number of fecal pen scores scored on the pen level. Results are shown as mean±sd across individual pigs or pens.

| **A** | *Individual level* | | | *Round 1* | *Round 2* |
| --- | --- | --- | --- | --- | --- |
| *Individual behavioral scoring* | *Proportion lying* | *Total* | | 0.76±0.05 | 0.67±0.06 |
|  |  | *Pen 1* | | 0.73±0.03 | 0.70±0.06 |
|  |  | *Pen 2* | | 0.73±0.04 | 0.62±0.07 |
|  |  | *Pen 3* | | 0.76±0.03 | 0.64±0.02 |
|  |  | *Pen 4* | | 0.82±0.02 | 0.72±0.04 |
|  | *Proportion standing* | *Total* | | 0.16±0.04 | 0.19±0.05 |
|  |  | *Pen 1* | | 0.15±0.03 | 0.17±0.04 |
|  |  | *Pen 2* | | 0.17±0.04 | 0.23±0.07 |
|  |  | *Pen 3* | | 0.18±0.03 | 0.21±0.02 |
|  |  | *Pen 4* | | 0.12±0.02 | 0.15±0.02 |
|  | *Proportion eating* | *Total* | | 0.05±0.02 | 0.07±0.02 |
|  |  | *Pen 1* | | 0.06±0.02 | 0.06±0.01 |
|  |  | *Pen 2* | | 0.06±0.01 | 0.07±0.02 |
|  |  | *Pen 3* | | 0.04±0.01 | 0.08±0.01 |
|  |  | *Pen 4* | | 0.03±0.01 | 0.07±0.02 |
|  | *Proportion walking* | *Total* | | 0.02±0.011 | 0.04±0.014 |
|  |  | *Pen 1* | | 0.03±0.007 | 0.05±0.010 |
|  |  | *Pen 2* | | 0.02±0.009 | 0.05±0.016 |
|  |  | *Pen 3* | | 0.02±0.004 | 0.04±0.017 |
|  |  | *Pen 4* | | 0.01±0.004 | 0.04±0.016 |
|  | *Proportion drinking* | *Total* | | 0.006±0.004 | 0.009±0.005 |
|  |  | *Pen 1* | | 0.011±0.003 | 0.011±0.006 |
|  |  | *Pen 2* | | 0.006±0.003 | 0.007±0.004 |
|  |  | *Pen 3* | | 0.005±0.004 | 0.009±0.005 |
|  |  | *Pen 4* | | 0.003±0.003 | 0.008±0.005 |
|  | *Proportion other total* | | | 0.01±0.006 | 0.02±0.010 |
| *Fecal swab scoring* | *N aberrant fecal swab color scores* | | *Total* | 13 | 19 |
|  |  |  | *Day 1* | 1 | 5 |
|  |  |  | *Day 3* | 3 | 6 |
|  |  |  | *Day 5* | 8 | 5 |
|  |  |  | *Day 8* | 1 | 3 |
|  |  |  | *Pen 1* | 4 | 2 |
|  |  |  | *Pen 2* | 3 | 10 |
|  |  |  | *Pen 3* | 5 | 5 |
|  |  |  | *Pen 4* | 3 | 4 |
|  | *N aberrant fecal swab consistency scores* | | *Total* | 19 | 21 |
|  |  |  | *Day 1* | 3 | 1 |
|  |  |  | *Day 3* | 2 | 1 |
|  |  |  | *Day 5* | 12 | 14 |
|  |  |  | *Day 8* | 2 | 5 |
|  |  |  | *Pen 1* | 6 | 5 |
|  |  |  | *Pen 2* | 4 | 6 |
|  |  |  | *Pen 3* | 5 | 5 |
|  |  |  | *Pen 4* | 6 | 6 |

|  |  | |  |  |  |  |
| --- | --- | --- | --- | --- | --- | --- |
| **B** | *Pen level* | | | *Round 1* | *Round 2* | *Round 3* |
| *Pen level activity* | *Percentage inactivity* | *Total* | | 92.1±3.57% | 89.5±1.86% | 88.0±1.98% |
|  |  | *Pen 1* | | 87.8% | 89.2% | 89.2% |
|  |  | *Pen 2* | | 92.0% | 88.7% | 87.3% |
|  |  | *Pen 3* | | 93.4% | 89.1% | 86.9% |
|  |  | *Pen 4* | | 95.2% | 91.1% | 88.5% |
|  | *Percentage eating* | *Total* | | 5.19±2.48% | 6.71±2.51% | 8.39±1.61% |
|  |  | *Pen 1* | | 7.12% | 7.19% | 7.57% |
|  |  | *Pen 2* | | 5.75% | 6.62% | 7.69% |
|  |  | *Pen 3* | | 4.48% | 7.21% | 9.53% |
|  |  | *Pen 4* | | 3.41% | 5.83% | 8.78% |
|  | *Percentage drinking* | *Total* | | 1.25±1.46% | 1.39%±0.98% | 1.45±1.18% |
|  |  | *Pen 1* | | 2.69% | 1.36% | 0.92% |
|  |  | *Pen 2* | | 0.87% | 1.39% | 1.93% |
|  |  | *Pen 3* | | 0.90% | 1.57% | 1.75% |
|  |  | *Pen 4* | | 0.55% | 1.24% | 1.21% |
|  | *Percentage moving* | *Total* | | 1.47±0.82% | 2.40±0.90% | 2.18±1.02% |
|  |  | *Pen 1* | | 2.40% | 2.27% | 2.27% |
|  |  | *Pen 2* | | 1.43% | 3.32% | 3.07% |
|  |  | *Pen 3* | | 1.25% | 2.14% | 1.82% |
|  |  | *Pen 4* | | 0.80% | 1.86% | 1.56% |
| *Fecal pen scoring* | *N aberrant fecal pen color scores* | *Total* | | 1 | 16 | 11 |
|  |  | *Period 1*  *(day 4-5)* | | 0 | 7 | 1 |
|  |  | *Period 2*  *(day 6-8)* | | 1 | 9 | 10 |
|  |  | *Pen 1* | | 0 | 4 | 4 |
|  |  | *Pen 2* | | 0 | 5 | 3 |
|  |  | *Pen 3* | | 1 | 4 | 3 |
|  |  | *Pen 4* | | 0 | 3 | 1 |
|  | *N aberrant fecal pen consistency scores* | *Total* | | 6 | 19 | 14 |
|  |  | *Period 1*  *(day 4-5)* | | 1 | 8 | 2 |
|  |  | *Period 2*  *(day 6-8)* | | 5 | 11 | 12 |
|  |  | *Pen 1* | | 2 | 5 | 3 |
|  |  | *Pen 2* | | 2 | 5 | 3 |
|  |  | *Pen 3* | | 2 | 5 | 4 |
|  |  | *Pen 4* | | 0 | 4 | 4 |
